# Protocol to investigate the effects of localized gene expression disruption on mouse limb development via direct siRNA injection and explant culture

**DOI:** 10.1016/j.xpro.2026.104402

**Published:** 2026-02-28

**Authors:** Ji-Hye Yea, Sharon E.S. Onggo, Suhani Dewagan, Kaiwen Chen, Kaily K. Young, Patrick Cahan

**Affiliations:** 1 Department of Biomedical Engineering, Johns Hopkins University School of Medicine, Baltimore, MD 21205, USA; 2 Institute for Cell Engineering, Johns Hopkins University School of Medicine, Baltimore, MD 21205, USA; 3 Krieger School of Arts and Sciences, Johns Hopkins University, Baltimore, MD 21218, USA

**Keywords:** developmental biology, Stem cells, tissue Engineering

## Abstract

Here, we present a protocol for localized gene knockdown in embryonic mouse hindlimb explants using direct small interfering RNA (siRNA) injection, combined with a semi-quantitative morphology scoring system to assess developmental outcomes. We describe steps for mating of samples, preparing hindlimb explants for culture and siRNA-Lipofectamine complex, and direct injection of the siRNA-Lipofectamine complex. We then detail procedures for explant culture and maintenance, data analysis, tissue fixation, and cryoprotection. This protocol approach enables spatially precise modulation of gene expression during limb and joint development.

## Highlights

• Steps for direct injection of gene expression modulators into embryonic limb explants
• Guidance on explant culture and localized gene modulation
• Instruction for semi-quantitative scoring of limb and joint morphology

## Abstract (editor highlights)

Publisher’s note: Undertaking any experimental protocol requires adherence to local institutional guidelines for laboratory safety and ethics.

## Graphical abstract

**Figure:**
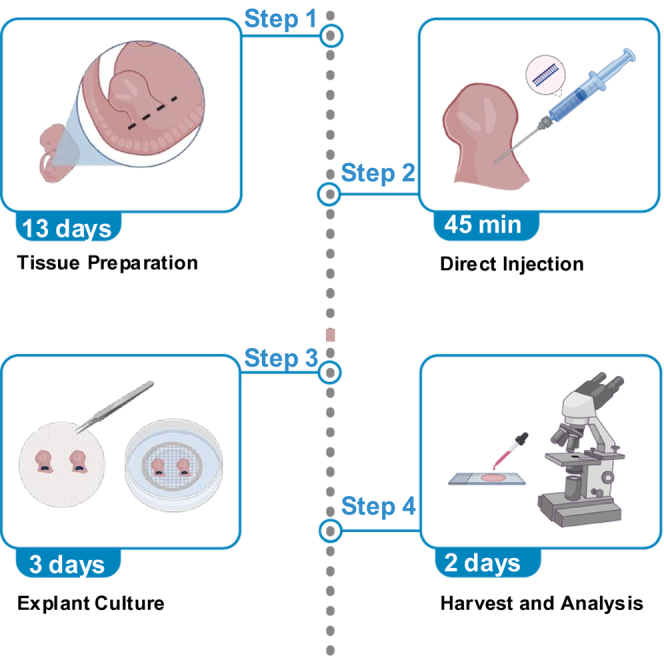

## Before you begin

**Figure 1:**
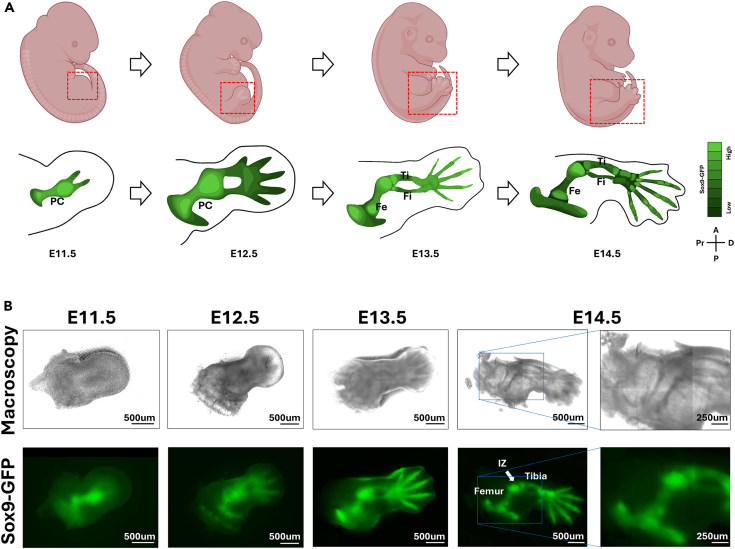
Overview of hindlimb skeletal development from E11.5 to E14.5 (A) Schematic diagram shows progressing development of the embryonic mouse hindlimb from E11.5 to E14.5. Sox9^+^ skeletal elements gradually expand and segment into recognizable skeletal structures, including the tibia and femur cartilage templates. (B) Macroscopic images and Sox-GFP fluorescence show normal hindlimb development, consistent with schematic diagram. PC, pre-cartilage condensation; IZ, Interzone; Ti, Tibia; Fe, Femur; Fi, Fibula; Pr, Proximal; D, Distal; A, Anterior; P, Posterior.

This protocol describes the use of direct injection for localized delivery of gene modulators into specific regions of embryonic rodent limbs during embryonic development. The protocol outlined below is optimized for injecting siRNA into the presumptive femur region of E12.5 embryonic mice, presenting a pre-cartilaginous stage (Figure 1).^1^ We use Sox9-IRES-EGFP knock-in (Sox9IE) mice (B6;129S4-Sox9tm1.1Tlu/J, JAX stock #030137, RRID: IMSR_JAX:030137) embryos because morphological development of the cartilage templates and synovial joints can be quantified with GFP signal, as Sox9 is a master transcription factor required for chondrogenic condensation and skeletal element formation during limb development.^2^^,^^3^^,^^4^ However, with appropriate adaptions, the system is likely to be applicable in other contexts, such as other limb regions, different mouse strains, other methods to alter gene expression, and other modes of perturbation (e.g., small molecules or signaling pathway modifiers).

Because tissue size, developmental timing and gene expression dynamics vary between strains and targets, optimization of injection parameters (e.g., timing, volume, concentration) is recommended for each gene modulator and model system before initiating large-scale experiments.

### Innovation

This protocol introduces a localized gene modulation strategy for embryonic mouse limb development that overcomes the spatial and temporal limitations of conventional genetic models, which often lack precise region-specific control and require extensive breeding schemes.^5^ By combining direct siRNA microinjection with ex vivo hindlimb explant culture, it enables region-specific gene knockdown within defined skeletal elements such as the femur or presumptive joint region. Compared to transgenic or conditional knockout approaches, this system provides a rapid, cost-effective, and flexible method to test gene function without extensive breeding or germline modification. The use of Sox9-IRES-EGFP reporter mice allows real-time visualization of chondrogenic condensation and patterning, confirming localized effects with minimal diffusion beyond the injection site. Furthermore, we developed an eight-parameter semi-quantitative morphological scoring system that standardizes phenotypic evaluation across experiments, facilitating reproducibility and comparison between perturbations. Collectively, this protocol integrates spatial precision, quantitative assessment, and visual monitoring into a single workflow, advancing studies of limb and joint development by making localized genetic perturbation broadly accessible.

### Institutional permission

The Institutional Animal Care and Use Committees at the Johns Hopkins University have approved all animal procedures utilized in this study.

**Figure 2:**
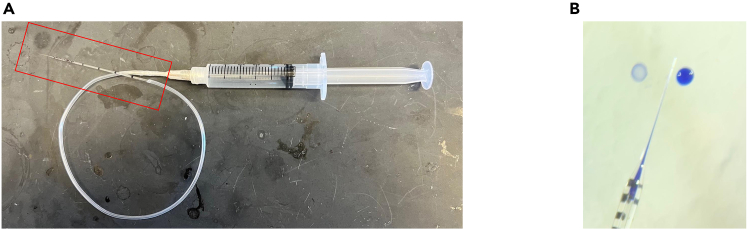
Syringe setup used for microinjection (A) Syringe setup with glass microneedle for microinjection (the red box indicates a fabricated glass microneedle). (B) Trypan blue was used to confirm injection volume, One tick is 90 μm.

### Mouse housing

**Timing: Variable**

1. House mice in an accredited animal facility under standard conditions: 40%–70% relative humidity, temperature of 22 °C, and a 12-hour light/dark cycle.
2. Provide ad libitum access to food and water, and include supply environmental enrichment items (e.g., tunnels, gnawing sticks) to promote animal welfare.

### Preparation of the microinjection syringe with micro-needle

**Timing: 10 min**

3. Fabricate glass micropipettes using a micropipette puller.
  a. Pull capillary glass tubes bidirectionally to form two tapered tips.
  b. Fracture the sealed tip gently using sterile forceps to create an open channel.
4. Attach the broken-tip micropipette to a 5 μL syringe via flexible tubing.
5. Seal the junction between the syringe and tubing using Parafilm or equivalent to prevent leakage (Figure 2A).

***Note:*** Confirm flow and suction by drawing trypan blue into the needle as a visual tracer (Figure 2B).

### Preparation of the support mesh for explant culture

**Timing: 1 min**

6. Cut the stainless-steel mesh into a 2.2 cm x 2.2 cm square.

### Sterilization of instruments

**Timing: 60 min**

7. Autoclave surgical instruments (e.g., scissors, forceps) and stainless-steel mesh using an autoclave at 121°C for 60 minutes before use.

**CRITICAL:** Ensure all tools remain sterile throughout dissection and injection steps to minimize contamination risk.

### Preparation of explant culture dishes

**Timing: ∼30 min**

8. BGJb medium supplemented with 1% Antibiotic-Antimycotic (Complete medium without serum, CM) at 37 °C for 20 minutes.
9. Pre-warm PBS at 37 °C for 20 minutes.
10. In a sterile organ culture dish with a center well:
  a. Add 2 mL of pre-warmed saline to the outer moat.
  b. Add 2 mL of pre-warmed CM to the center well.
11. Place a sterilized, pre-cut stainless-steel mesh over the center well to support the tissue/filter.

***Note:*** To maintain sterility, briefly immerse the mesh in 70% ethanol if contamination is suspected, and let it fully air-dry before use.

## Key resources table

**Table:** 

REAGENT or RESOURCE	SOURCE	IDENTIFIER
**Chemicals, peptides, and recombinant proteins**		

Sox9 silencer siRNA	Thermo Fisher Scientific	s74193
Negative siRNA	Thermo Fisher Scientific	4390843
Silencer™ Select Negative Control No. 1 siRNA	Thermo Fisher Scientific	4390771
Lipofectamine RNAiMAX Transfection Reagent	Thermo Fisher Scientific	13778100
BLOCK-iT™ Alexa Fluor™ Red Fluorescent Control	Thermo Fisher Scientific	14750100

**Other**		

Sutter P-97 Flaming/Brown Type Micropipette Puller	Automate Scientific	SKU: SU-P-97
OCT Compound for Cryostat Sectioning, Tissue-Tek®	Tedpella	27050
Parafilm™ M Laboratory Wrapping Film	Thermo Fisher Scientific	PM996
16% paraformaldehyde (PFA) Aqueous Solution, EM Grade	Electron Microscopy Sciences	15710
Stainless Steel Woven Wire 10 Mesh	TIMESETL	TXJ-277-US
Falcon® 60 mm TC-treated Center Well Organ Culture Dish	Falcon	353037
Permeable Support for 6-well Plate with 1.0 μm Transparent PET Membrane	Falcon	353102
Antibiotic-Antimycotic 100×	Thermo Fisher Scientific	15240062
Gibco BGJb medium	Thermo Fisher Scientific	12-591-038
5ml Syringe	Becton Dickinson	309646
60 mm x 15 mm Petri Dish	CELLTREAT	120423A001
Precision Calibrated Micropipettes, 1–5 μL, 1.092 O.D./0.3404I.D. (mm)	Drummond Scientific	2-000-001
Phosphate Buffered Saline (PBS) (1×), pH 7.2	Quality Biological	114-056-101
PrecisionGlide Needle Aiguille	Becton Dickinson	305186
Tygon ND-100-80 Tubing - 1.02 mm (0.04″) ID x 1.78 mm (0.07″) OD	Tubing	AD04127
15 Disposable Scalpels Sterile Surgical Blade	P&P	0197
Sharp-Pointed Dissecting Scissors	Thermo Fisher Scientific	08–935
Castroviejo Micro Scissors 3.5	A2Z SCILAB	686797873241
Domont Tweezer	ROBOZ	RS-5040
Cole-Parmer Scissor, 160 mm	Cole-Parmer	UX-06287-20
KIMwipes	KimberlyClark Science	06-666
Parafilm™ M Laboratory Wrapping Film, 4″ Width, 125 ft/roll	Thermo Fisher Scientific	PM996
Stereo microscope	ZEISS	Stemi 508

## Step-by-step method details

### Timed mating for E12.5 samples

**Timing: 13 days**

This step outlines timed mating procedure for setting up mouse mating to obtain embryos at embryonic day 12.5 (E12.5). Accurate timing is essential for developmental consistency.

1. Pair one male mouse with two female mice in the late afternoon.
2. After 2 hours of co-housing, check for the presence of vaginal plugs to confirm mating and separate plugged females.
3. House plugged females individually until embryonic day 12.5.
4. Euthanize pregnant females at E12.5 by CO_2_ asphyxiation.

***Note:*** To ensure accurate staging, monitor for vaginal plugs within a limited post-cohousing window. Delayed plug checks may compromise staging accuracy.

### Preparation of hindlimb explants for culture

**Timing: ∼30 min**

This step describes the dissection and isolation of E12.5 embryonic hindlimbs for ex vivo culture in a sterile hood.

**Figure 3:**
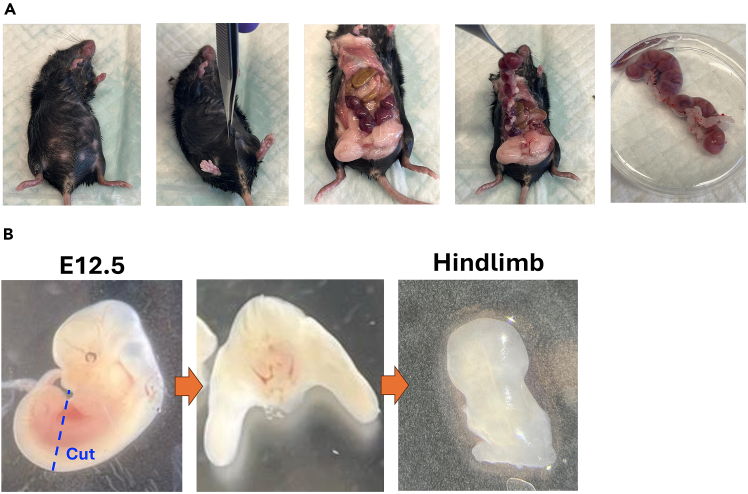
Isolation of E12.5 hindlimbs from pregnant mice (A) Harvest of E12.5 embryos from pregnant female. (B) Dissection of hindlimbs and isolation from an embryo body.

5. Disinfect the dissection surface with 70% ethanol and line with an absorbent pad in sterile hood.
6. Place the euthanized pregnant mouse supine on the clean surface.
7. Disinfect the abdominal skin using 70% ethanol.
8. Lift the skin with forceps and make an incision through the abdominal wall to expose the uterus.
9. Excise the uterine horn and place it immediately into PBS (Figure 3A).
10. Wash the uterus twice in fresh PBS to remove blood and debris.
11. Isolate embryos from the uterine tissue using fine scissors under PBS.
12. Transfer embryos into a clean dish containing fresh PBS.
13. Place samples under a dissection microscope.
14. Make a transverse incision at the lumbar region with a #15 blade; collect the lower body with hindlimbs.
15. Separate each hindlimb at the hip joint using a scalpel (Figure 3B).
16. Place hindlimbs onto a filter, and transfer to a Petri dish for injection.

***Note:*** Minimize tissue handling time and avoid direct pinching of the tissue; use gentle scooping or sliding motions to preserve tissue integrity.

### Preparation of the siRNA-Lipofectamine complex

**Timing: 5–10 min**

This step describes the preparation of a siRNA-Lipofectamine RNAiMAX complex immediately prior to injection.

17. Combine 1 μL of Sox9 siRNA (100 pmol/μL), negative control siRNA, or Fluorescent control with 2 μL of Lipofectamine RNAiMAX (total volume is 3 μL) in a sterile microcentrifuge tube.
18. Gently pipette mix without vortexing.

***Note:*** In this protocol, siRNA and Lipofectamine RNAiMax are directly mixed without prior dilution in OPTIMEM. This was implemented to minimize the total injection volume required for localized delivery in embryonic hindlimb explants.

**CRITICAL:** Prepare and use the complex after 5 mins after preparation and do not let it sit for a long time to prevent aggregation. Avoid freeze-thaw cycles of siRNA stocks to maintain knockdown efficiency.

### Direct injection of the siRNA-Lipofectamine complex

**Timing: 5 min**

This step describes the localized delivery of siRNA into the femoral region of hindlimb explants.

19. Remove residual PBS by gently blotting the explant samples using a KimTech wipe.

***Note:*** Residual fluid may cause the sample to float, resulting in targeting inaccuracy.

**Figure 4:**
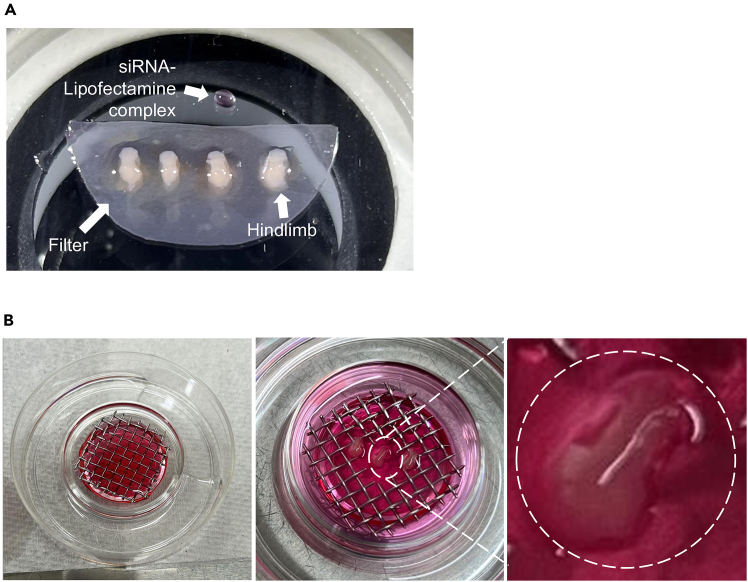
Preparation of siRNA complex and explant culture setup (A) Preparation of siRNA complex and hindlimbs in a filter sheet. (B) Placement of explants on stainless steel mesh for air-medium interface culture.

20. Prepare 1 μL of siRNA-Lipofectamine complex in the same Petri dish (Figure 4A).
21. Load 100 nL of the solution into the syringe with micro-needle under the microscope.
22. Hold the micro-needle parallel to the tissue surface for stable insertion.
23. Gently insert the needle into the target site and inject full 100 nL per hindlimb explant slowly while withdrawing.

**CRITICAL:** Inject slowly to prevent tissue. Fast injection may perforate tissue and cause leakage.

24. Allow the complex to diffuse for 30 seconds.
25. Rinse the explant samples gently with PBS to remove any leaked material.
26. Transfer the filter with injected tissue back onto the mesh in the prepared culture dish (Figure 4B).
27. Perform a second injection on day 1, if needed.

***Note:*** A second injection may be required if the expression of the target gene, which was initially reduced by siRNA treatment, shows recovery during the culture period.

***Note:*** Adjust injection volume and frequency based on the anatomical target and desired knockdown duration.

**Figure 5:**
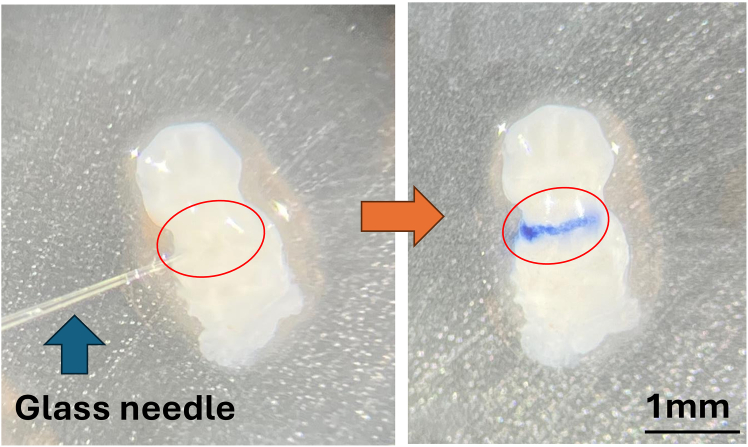
Injection of trypan blue into hindlimb explant indicating minimal diffusion

***Note:*** To assess the spatial distribution of the injected materials, we injected trypan blue dye using the same procedures as the siRNA delivery.^6^ After injection and following two saline washes to remove excess dye, we found that the trypan blue remained confined to the femoral injection site after 2 h (Figure 5).

### Explant culture and maintenance

**Timing: 3 days**

This step describes the maintenance of cultured hindlimbs over three days under static conditions with daily repositioning and media replacement.

28. Gently reposition explants daily using fine forceps to prevent adhesion to the filter.
29. Carefully remove the spent culture medium by gently inserting a pipette tip through the gaps of the stainless-steel mesh, taking care not to disturb the filter, and replace it with fresh CM daily.
30. Continue culture maintenance for the full 72-hour period.

***Note:*** Although hindlimb explants can be maintained for up to 7 days under these culture conditions, all experiments in this study were performed within a 3-day culture period.

During culture, the hindlimb can be directly imaged for morphological changes under brightfield and GFP fluorescence in EVOS fluorescence microscope (Thermo Fisher Scientific).

**Figure 6:**
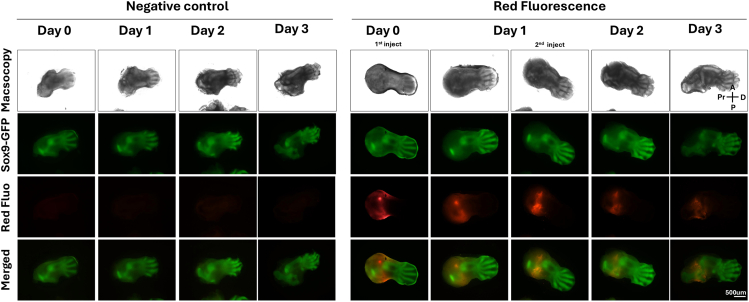
Localization of local siRNA injection targeting the femur Time-course images shows the localization of injected Bock-iT fluorescent oligos. The red fluorescence remains confined to the femoral region for three days post-injection, indicating localized uptake and limited diffusion. Red Fluo.: Block-iT red signal. Pr, Proximal; D, Distal; A, Anterior; P, Posterior.

***Note:*** To track the injected siRNA, it is recommended to use a siRNA-Lipofectamine complex with a fluorescence control, Block-iT Alexa Fluor Red Fluorescent Control (Thermo Fisher Scientific). Fluorescence imaging shows that the red signal remains sharply confined to the injection site for day 3, with minimal lateral diffusion^7^ (Figure 6).

**Figure 7:**
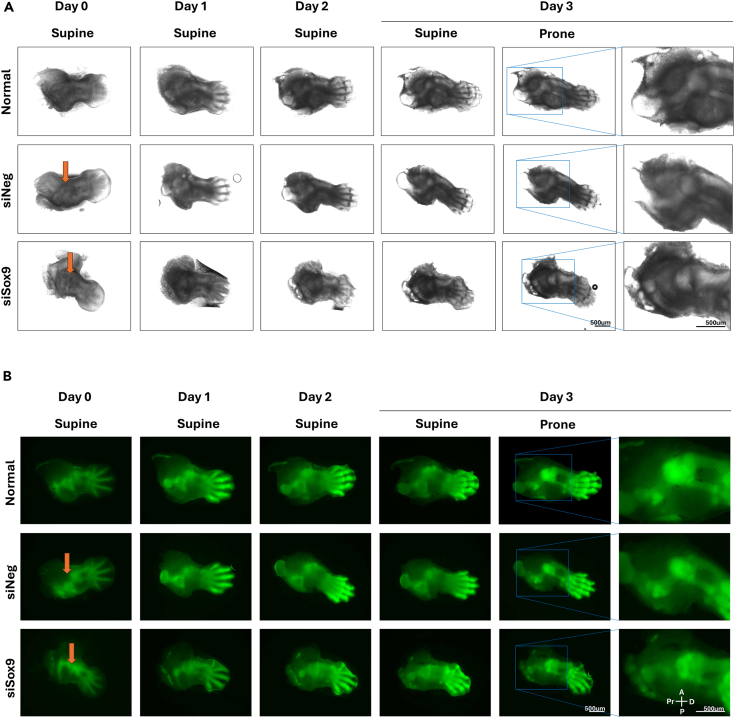
Morphogenetic effects of local siRNA injection targeting the femur Macroscopic (A) and Sox9-GFP fluorescence (B) images of explants cultured for three days after siRNAs treatments. Normal and siNeg groups exhibit progressive femoral elongation and structural refinement, but siSox9 group shows disrupted femoral morphogenesis, particularly in the midshaft region. Pr, Proximal; D, Distal; A, Anterior; P, Posterior.

***Note:*** During the culture period, the knockdown effect of siRNA can be monitored in real time. Femur-specific development defects following siSox9 injection into femur can be examined. Macroscopic imaging showed that tibia formation began around day 1 and progressed normally in all groups through day 3, with well-defined skeletal structures and strong GFP expression. Moreover, control and siNeg group exhibited progressive femoral elongation and morphological refinement between day 1 and day 3.^8^ However, siSox9-treated limbs failed to elongate properly, particularly in the femur body region. So, in the siSox9 group the femur was underdeveloped and/or obscured by the pelvis, indicating disrupted femoral morphogenesis (Figure 7).

### Semi-quantitative and quantitative data

**Timing: 20 min**

We developed an 8-parameter semi-quantitative grading system (scores ranging from 0 to 3 per category; maximum score = 24) to assess developmental outcomes based on femoral morphology and joint shape (Table 1).

31. Using the macroscopic and fluorescence images, semi-quantitative scoring is performed according to the criteria in Table 1.

***Note:*** “Appears normal” indicates qualitative evaluation based on visual inspection.

32. Open fluorescence images from Sox9-GFP-expressing Tibia and Femur in imageJ (NIH).

***Note:*** Quantitative measurements were performed using ImageJ (NIH). Length was measured by tracing the longitudinal axis of the tibial element, and thickness was measured along the transverse axis at the midpoint of the skeletal element. Area was defined by manually outlining the region of interest based on Gdf5 signaling boundaries. Signal density was calculated as the mean fluorescence intensity within the defined area, normalized to the measured area.

33. Measure length and area as well as fluorescence intensity.

**Figure 8:**
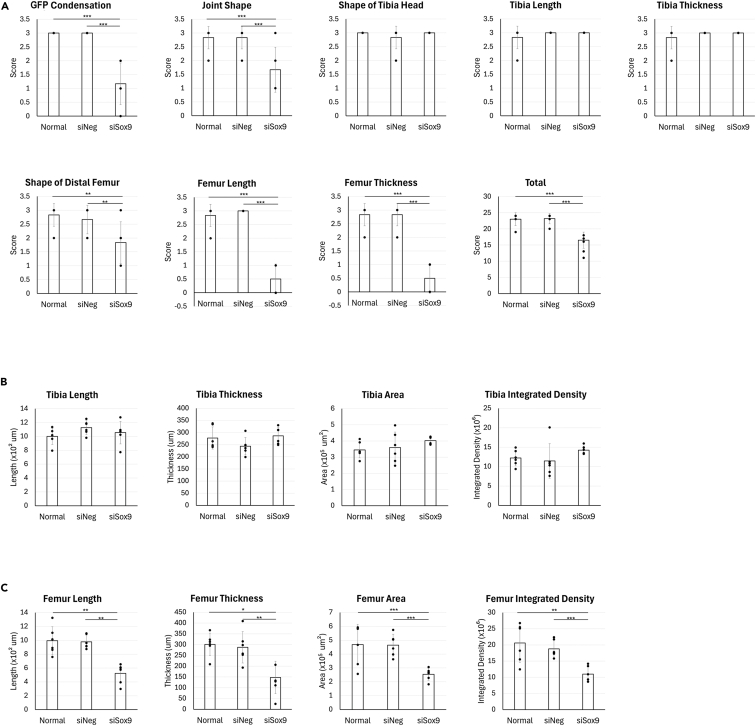
Quantitative and semi-quantitative evaluation of femoral development (A) Semi-quantitative scoring of eight parameters related to tibia, femoral and joint morphology, as well as GFP condensation shows significantly lower scores in femur-related features in siSox9 group compared to the Normal and siNeg groups. (B and C) Quantitative measurements using ImageJ were performed on tibia and femoral regions. Tibia-related values remain unchanged, but femur-related values are significantly reduced in siSox9 group compared to the Normal and siNeg groups. n = 6 was used for analysis. Pr, Proximal; D, Distal; A, Anterior; P, Posterior. All data are expressed as means, and each data point represents and individual limbs. One-way ANOVA with Tukey’s multiple-comparisons test was used for statistical analysis. ^∗^P < 0.05, ^∗∗^P < 0.01 and ^∗∗∗^P < 0.001.

***Note:*** This system enables assessment of gene knockdown effects on limb development through both semi-quantitative and quantitative analyses. In semi-quantitative scoring, siSox9-injected limbs can be observed to exhibit lower scores for GFP signal intensity, joint morphology, femur shape, length, and thickness compared to control and siNeg groups (Figure 8A). Quantitative analysis further allows comparison of skeletal structural parameters. It shows that while tibial area, thickness, and integrated density remain unchanged among groups, siSox9-treated limbs display significantly reduced femoral area, length, thickness, and integrated density, confirming the spatial specificity and efficacy of the knockdown (Figures 8B and 8C).

**Table 1:** Morphological scoring system for joint and limb development

Parameter	Criteria	Score
Sox9-GFP condensation	Sox9-GFP signal clearly reflects anlagen shape	3
	Sox9 signaling does not clearly show the bone shape	2
	Sox9 signaling appears dispersed or reduced	1
	Sox9 signaling is barely visible	0
Joint Shape	The tibia and femur are clearly separated and the joint shape appears normal	3
	The joint appears to be formed but its shape is not normal	2
	The tibia and femur are not clearly separated or the shape is severely abnormal	1
	The joint (JT) is not formed or appears fused	0
Shape of Tibia Head	The tibial head shows visible curvature (condyle shape) and appears normal	3
	The tibial head curvature (condyle shape) is not visible or changed	2
	The tibial head appears unclear or severely changed	1
	The head shape is completely absent	0
Tibia Length	The length of the tibia appears normal	3
	The tibia is shortened to two-thirds	2
	The tibia is shortened to one-third	1
	The tibia is shortened to less than one-third or is barely visible	0
Tibia Thickness	The tibial thickness appears normal	3
	The tibia appears two-thirds thinner	2
	The tibia appears one-third thinner	1
	The tibia is less than one-third in thickness or barely visible	0
Shape of Distal Femur	The distal femur shows a distinct convex curvature and appears normal	3
	The convex curvature of the distal femur is not visible or changed	2
	The distal shape of the femur is unclear or severely changed	1
	The distal femoral shape is completely absent	0
Femur Length	The length of the femur appears normal	3
	The femur is shortened to two-thirds	2
	The femur is shortened to one-third	1
	The femur is shortened to less than one-third or is obscured by the pelvic bone at the joint	0
Femur Thickness	The femoral thickness appears normal	3
	The femur appears two-thirds thinner	2
	The femur appears one-third thinner	1
	The femur is less than one-third in thickness or barely visible	0

### Tissue fixation and cryoprotection

**Timing: 1 day**

This step outlines fixation and sucrose-based cryoprotection of tissues for downstream analysis.

34. Fix explant tissues on the filter in 4% paraformaldehyde at 4 °C for 4 hours.
35. Wash samples three times with PBS at 4 °C for 10 minutes each per wash.
36. Sequentially incubate the tissues in sucrose solution at 4 °C; 10% sucrose for 2 hours, 20% sucrose for 2 hours, 30% sucrose for 12 hours (or overnight).
37. Proceed with embedding and sectioning for downstream analysis.

***Note:*** Keep explant tissues on the filter throughout fixation and cryoprotection to preserve anatomical orientation.

## Expected outcomes

This protocol enables localized knockdown of gene expression in the developing mouse hindlimb through direct siRNA injection with micro-needle into a specific anatomical region within an explant culture system.^9^^,^^10^ The explant system supports continued in vitro development of isolated organs, providing a controlled environment in which gene modulators can be applied to targeted regions at specific developmental time points.^11^^,^^12^^,^^13^

In our system, injected siRNA remains largely confined to the target site, typically the middle femur anlage, with limited diffusion to surrounding tissues, for three days of culture post injection. Delivering siRNA via direct injection does not alter the overall trajectory of ex vivo limb development. During the culture period, Sox9 expression can be monitored in real time using Sox9-IRES-EGFP reporter mice, in which EGFP is knocked into the 3′ UTR of the Sox9 locus. This strategy allows tracking of Sox9 expression without affecting normal limb development. This enables direct observation of developmental responses to siRNA-mediated gene modulation.

The degree of target gene knockdown may vary depending on siRNA design and cellular uptake efficiency. In our study, siSox9 injection led to an approximately 50% reduction in Sox9 expression in the femur region, as estimated by GFP signal intensity.

Therefore, this system provides a powerful and accessible platform for studying region-specific gene function in joint and skeletal development.

## Limitations

This method offers a powerful tool for spatially precise gene knockdown during limb development, but some key limitations should be considered. Because embryonic limbs are very small and fragile, the injection procedure requires significant manual dexterity. Minor differences in injection depth, angle, or placement can lead to variability in siRNA delivery and knockdown efficiency, especially for inexperienced users. We recommend practicing with visual tracers such as trypan blue or fluorescent control oligos, and using micromanipulators to improve consistency.

Additionally, embryos from the same litter can differ by several hours in developmental stage, which may influence their response to gene modulation. Careful staging and standardized dissection are important to reduce this biological variability.

This ex vivo culture system supports cartilage development but does not fully reproduce the complexity of in vivo limb development, such as the formation of vasculature, nerves, and muscle. Also, systemic cues present in whole animals are absent. Therefore, researchers should assess whether this model is suitable for their specific tissue during development.

Embryonic development can vary both between litters and within the same litter due to differences in fertilization and implantation timing. To minimize variability, we recommend selecting embryos based on distal limb morphology (e.g., paddle-shaped buds), rather than assuming uniform staging across a litter.^14^

Finally, this protocol was optimized using Sox9-GFP reporter mice. Its use in other strains or without fluorescent reporters is untested, and phenotypic assessment may be more subjective. In the future, machine learning applied to brightfield images may improve objectivity and broaden applicability across different models.

## Troubleshooting

### Problem 1

Tissue tearing or collapse during handling (related to Step 6–16).

### Potential solution

Avoid pinching the tissue directly with forceps. Instead, use the side of the forceps to slide to the tissue or scoop it gently.

### Problem 2

Curling or distortion of the samples after fixation (related to Step 34–37).

### Potential solution

To minimize disturbance, avoid pouring solutions directly onto the sample. Instead, gently deliver fixation and sucrose solutions along the wall of the container. Ensure that the sample remains securely attached to the filter membrane throughout all steps for the fixation and sucrose process.

### Problem 3

Difficulty inserting the needle into tissue (related to Step 19–23).

### Potential solution

Fabricate the needle with a tapered, beveled tip, similar to a conventional injection needle, rather than a blunt or rounded shape. This improves tissue penetration. Remove excess saline or CM from the surface of both the tissue and the filter using a wiper prior to injection.

### Problem 4

Contamination during explant culture (related to all step).

### Potential solution

Ensure that all tools and materials (e.g., mesh, forceps, scissors) are properly sterilized using the autoclaving or ethanol treatment prior to use.

Avoid prolonged exposure of culture dishes to ambient air. Minimize lid opening time during injection and media replacement.

### Problem 5

Tissues adhere to each other during culture (related to Step 28–30).

### Potential solution

When placing multiple explants on the same filter, ensure sufficient spacing between samples to prevent contact during incubation.

Do not overfill the culture dish, when adding CD, avoid levels that submerge the mesh or cause the tissue to float.

### Problem 6

No morphological changes are observed following direct injection of known regulator of limb development (related to Step 19–23).

### Potential solution

Validate siRNA efficacy in cultured primary cells prior to injection. Efficacy can vary based on probe design and lot to lot differences. Aliquot siRNA to minimize multiple freeze thaw cycles.

## Resource availability

### Lead contact

Further information and requests for resources and reagents should be directed to and will be fulfilled by the lead contact, Patrick Cahan (patrick.cahan@jhmi.edu).

### Technical contact

Technical questions on executing this protocol should be directed to and will be answered by the technical contact, Ji-Hye Yea (jyea2@jh.edu).

### Materials availability

This study did not generate new unique reagents.

### Data and code availability

No new code was generated for these analyses. Primary data are shared by the lead contact upon request after publication.

## Acknowledgments

Research reported in this publication was supported by the 10.13039/100000057National Institute of General Medical Sciences of the 10.13039/100000002National Institutes of Health under award no. R35GM124725.

## Author contributions

Investigation and conceptualization, P.C. and J.-H.Y.; methodology, J.-H.Y.; writing – original draft, J.-H.Y.; writing – review and editing, all authors; visualization, J.-H.Y., S.E.S.O., K.C., S.D., and K.K.Y.; project administration, P.C.; funding acquisition, P.C.

## Declaration of interests

The authors declare no competing interests.
